# Supplementary material for: Transcription Attenuation in Synthetic Promoters in Nonoverlapping Tandem Formation
Source: Biochemistry. 2024 Jul 12;63(16):2009–22. doi: 10.1021/acs.biochem.4c00012 (PMC11339919; doi:10.1021/acs.biochem.4c00012)
Supplement: Supplementary file 1 — bi4c00012_si_001.pdf [file bi4c00012_si_001.pdf]

Supplementary material for "Transcription attenuation in synthetic promoters in non-overlapping tandem formation"

Vatsala Chauhan[1,2], Ines S. C. Baptista[1], Amir M. Arsh[1], Rahul Jagadeesan[1], Suchintak Dash[1], and Andre S. Ribeiro[1]

[1]Faculty of Medicine and Health Technology, Tampere University, 33520, Finland.

[2]Department of Cell and Molecular Biology (ICM), Uppsala University, 751 24, Sweden

Supplementary Sections

S1. Stochastic model of non-overlapping tandem promoters

S1.1. Modeling single gene expression with two-step transcription initiation, repression, and induction mechanisms

We modelled active transcription initiation in *E. coli* as a two-step stochastic process [1]. Reaction S1 models an RNAP finding a promoter and committing to transcription initiation (completing the closed complex formation) at the stochastic rate constant k_b. Reaction S2 models the RNAP escape from the promoter (Pro), at the rate k_{esc}. This step accounts for open complex formation and promoter escape, which completes the initiation process [2,3]. This step also frees the promoter for new RNAPs.

After escaping, the RNAP quickly elongates ($RNAP_e$) and assembles the RNA coded by the gene. In the end, the RNA and RNAP are released (reaction S3). The number of nucleotides of the gene, *No. nuc.*, was set to 890 to match the average gene length of *E. coli* [4]. Instead, one could have set it to 708 bp, which is the length of the mCherry coding region (Results section "Assembly of the synthetic, non-overlapping tandem promoters in single-copy plasmids."), but this would not substantially affect the results.

Next, we model elongation as a single step, for simplicity (instead of multiple steps as in [5]). This does not influence mean elongation time lenght, only variance. We also included RNA degradation, in order to be able to estimate mean RNA numbers (reaction S4).

Finally, we added a repression mechanism. The repressors (Rep) binding rate to a free promoter is k_r and the unbinding rate is k_a (reactions S5 and S6). When Rep is bound, the promoter is unavailable to RNAP binding. The influence of repressors can be strongly reduced by inducers, *Ind*. These can directly bind/unbind repressors (at rates k_{ind} / k_{unind}) (reaction S7 and S8). When bound by an inducer, the repressor cannot bind to the promoter.

$$Pro + RNAP \xrightarrow{k_t} Pro.RNAP \quad \text{(S1)}$$

$$Pro.RNAP \xrightarrow{k_{esc}} Pro + RNAP_e \quad \text{(S2)}$$

$$RNAP_e \xrightarrow{k_{elong}/_{No.\ nuc.}} RNA + RNAP \quad \text{(S3)}$$

$$RNA \xrightarrow{k_d} \emptyset \quad \text{(S4)}$$

$$Pro + Rep \xrightarrow{k_r} Pro.Rep \quad \text{(S5)}$$

$$Pro.Rep \xrightarrow{k_a} Pro + Rep \quad \text{(S6)}$$

$$Ind + Rep \xrightarrow{k_{ind}} Ind.Rep \quad \text{(S7)}$$

$$Ind.Rep \xrightarrow{k_{unind}} Ind + Rep \quad \text{(S8)}$$

S1.2. Modeling non-overlapping tandem promoters with transcriptional interference due to RNAP collisions leading to fall-offs with repression and induction mechanisms.

To model non-overlapping tandem promoters, we adapted a model for operons with multiple promoters in tandem formation, recently published in [6]. First, we identify the promoters according to their position (Pro^U and Pro^D, where U and D stand for upstream and downstream, respectively). Similarly, we identify elongating RNAPs ($RNAP_e$) and RNAs, depending on which promoter they start from. Given this, Reactions S9 and S10 model transcription initiation of the downstream and upstream promoter, respectively, as follows:

$$Pro^D + RNAP \xrightarrow{k_t^D} Pro^D.RNAP \quad \text{(S9)}$$

$$Pro^U + RNAP \xrightarrow{k_t^U} Pro^U.RNAP \quad \text{(S10)}$$

Next, reaction S11 allows the RNAP at Pro^D.RNAP to escape the promoter, making it available for new transcription events. Afterwards, the RNAP can complete elongation and the RNA be released (reaction S12):

$$Pro^D.RNAP \xrightarrow{k_{esc}^D} Pro^D + RNAP_e^D \quad \text{(S11)}$$

$$RNAP_e^D \xrightarrow{k_{elong}/_{No.\ nuc.}} RNA^D + RNAP \quad \text{(S12)}$$

After forming Pro^U.RNAP complexes, the RNAP needs to escape the promoter (reaction S13):

$$Pro^U.RNAP \xrightarrow{k_{esc}^U} Pro^U + RNAP_e^U \quad \text{(S13)}$$

Then, the following events can occur to $RNAP_e^U$. First, if the downstream promoter is unoccupied, $RNAP_e^U$ can pass by and complete the RNA (reaction S14):

$$RNAP_e^U \xrightarrow{k_{elong}/_{No.\ nuc.}} RNA^U + RNAP \quad \text{(S14)}$$

Contrarily, if the downstream promoter is occupied by an RNAP, $RNAP_e^U$ will collide with it. In that case: (i) either $RNAP_e^U$ will fall-off (reaction S15); or (ii) both RNAPs fall-off (reaction S16):

$$RNAP_e^U + Pro^D.RNAP \xrightarrow{k_c \cdot f_1} Pro^D.RNAP + RNAP \quad \text{(S15)}$$

$$RNAP_e^U + Pro^D.RNAP \xrightarrow{k_c \cdot f_2} Pro^D + RNAP + RNAP \quad \text{(S16)}$$

S15 and S16 are competing reactions, and one of them has to occur. We control their mean rate using a base rate constant (k_c). Meanwhile, we control their relative frequency by multiply k_c with either f_1 or f_2, respectively, where: $f_1 + f_2 = 1$. Here, f_1 stands for the frequency with which only $RNAP_e^U$ falls-off, while f_2 stands for the frequency with which both RNAPs fall-off.

Noteworthy, at quasi-equilibrium, the RNAP collisions will affect mean RNA levels, only when causing RNAP fall-offs. Thus, since we are focusing on mean RNA levels, we do not model collisions that do not cause fall-offs. Also, we expect little differences in mean RNA levels at quasi-equilibrium between when the RNAP that falls-off is $RNAP_e^U$ or, instead, is the RNAP at the downstream promoter. Thus, we only model the first case (since the binding of $RNAP_e^U$ to DNA should be comparatively weaker [7]).

Finally, we modeled external repression and activation of the promoters. Reaction S17-S18 and S19-S20 model the binding of specific repressors, Rep^U and Rep^D, to the upstream and downstream promoters, respectively:

$$Pro^U + Rep^U \xrightarrow{k_r^U} Pro^U.Rep^U \quad \text{(S17)}$$

$$Pro^U.Rep^U \xrightarrow{k_a^U} Pro^U + Rep^U \quad \text{(S18)}$$

$$Pro^D + Rep^D \xrightarrow{k_r^D} Pro^D.Rep^D \quad \text{(S19)}$$

$$Pro^D.Rep^D \xrightarrow{k_a^D} Pro^D + Rep^D \quad \text{(S20)}$$

These repressors can be inactivated by the binding of their respective inducers, Ind^U and Ind^D, respectively (reaction S21-S22 for Rep^U and reaction S23-S24 for Rep^D), as follows:

$$Ind^U + Rep^U \xrightarrow{k_{ind}^U} Ind^U.Rep^U \quad \text{(S21)}$$

$$Ind^U.Rep^U \xrightarrow{k_{unind}^U} Ind^U + Rep^U \quad \text{(S22)}$$

$$Ind^D + Rep^D \xrightarrow{k_{ind}^D} Ind^D.Rep^D \quad \text{(S23)}$$

$$Ind^D.Rep^D \xrightarrow{k_{unind}^D} Ind^D + Rep^D \quad \text{(S24)}$$

Finally, we considered that, if the downstream promoter subject to repressors, the RNAP elongating from the upstream promoter could collide with a bound repressor. When occuring, similarly to when the downstream promoter is occupied by an RNAP, either $RNAP_e^U$ will fall-off (reaction S25, with frequency f_r) or the bound repressor will fall-off (reaction S26, with frequency (1- f_r)). For simplicitly, we do not model cases where both repressor and RNAP fall-off since, given the number of repressors and their binding and unbinding rate constants, this simplification did not influence the dynamics.

$$RNAP_e^U + \ Pro^D.Rep^D \xrightarrow{k_c \cdot f_r} Pro^D.Rep^D + RNAP \quad \text{(S25)}$$

$$RNAP_e^U + \ Pro^D.Rep^D \xrightarrow{k_c \cdot (1-f_r)} Pro^D + Rep^D + RNAP_e^U \quad \text{(S26)}$$

The RNAs produced in reactions S12 and S14 can decay via reactions S27 and S28, respectively:

$$RNA^U \xrightarrow{k_d} \emptyset \quad \text{(S27)}$$

$$RNA^D \xrightarrow{k_d} \emptyset \quad \text{(S28)}$$

Finally, all parameter values and supporting references are shown in Table S1.

Supplementary Figures

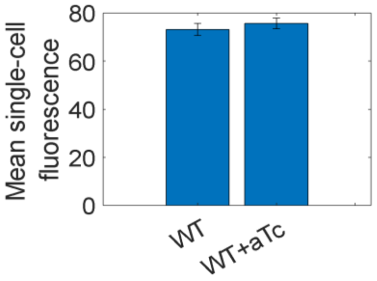

Figure S1: Single-cell fluorescence levels in arbitrary units. We used wild-type (WT) DH5α-Pro cells, absent of our plasmids, with and without the inducer, aTc. Fluorescence was measured 180 minutes (after adding aTc) at an $O.D._{600}$ of 0.3. The error bars represent the SEM from 3 biological replicates.

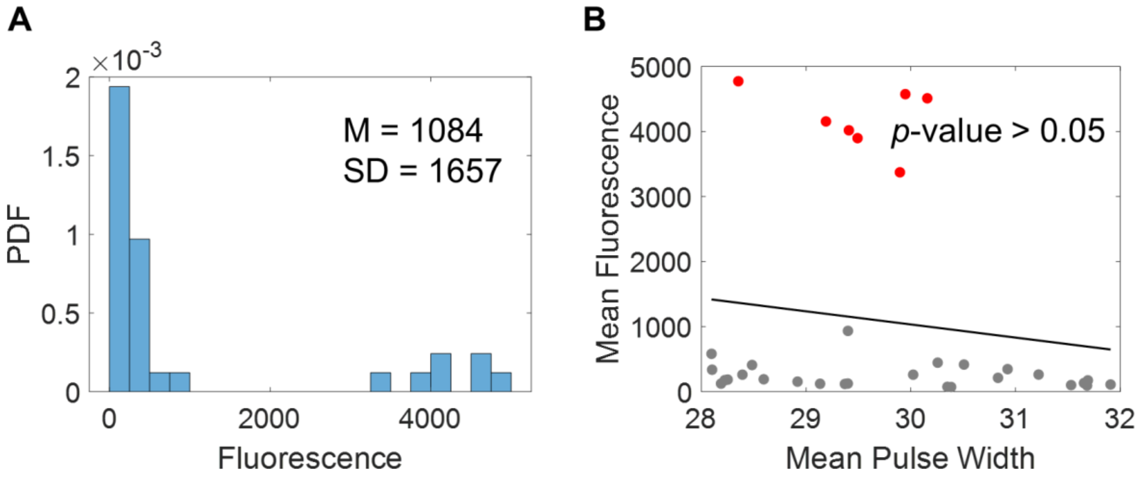

Figure S2: **(A)** Probability density function (PDF) of mean single-cell fluorescence levels. Also shown are the mean (M) and standard deviation (SD) of the distribution (top right). The SD is used as a threshold for the identification of outliers in Figure 2C in the main manuscript. **(B)** Scatter plot of the mean single-cell fluorescence plotted against the mean single-cell pulse width in each condition. The conditions are listed in Figure 2B in the main manuscript, including those classified as outliers (red balls). Also shown is a best fitting line. Visibly, the outliers (red points) affect the best-fitting line, causing it to not fit the bulk of the data points (grey). However, excluding them did not alter the conclusion, i.e., that one cannot conclude that the best fitting line fits better than a horizontal line (p-value > 0.05) (Methods section" Fitting and statistical analysis"). As such, we opted for not removing them from the line fitting.

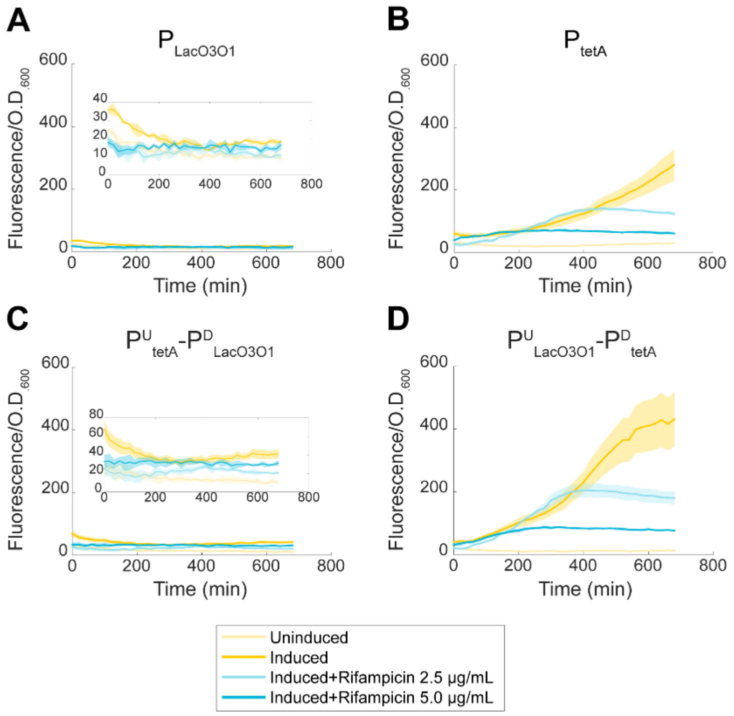

Figure S3: Average single-cell fluorescence levels of individual and non-overlapping tandem promoters. We measured population fluorescence levels (by spectrophotometry) and normalized them by the corresponding O.D.$_{600}$ to get a proxy for the average single-cell fluorescence levels (Methods section “Spectrophotometry”). Data for when uninduced, when fully induced (all promoters), and when fully induced while also subject to rifampicin (2.5 μg/mL and 5.0 μg/mL). The shaded areas represent the SEM from 3 biological replicates. “Induced” stands for when all promoters of the construct are fully induced. “Uninduced” stands for when no promoter is induced. The insets show the same data when zooming a smaller range of y values.

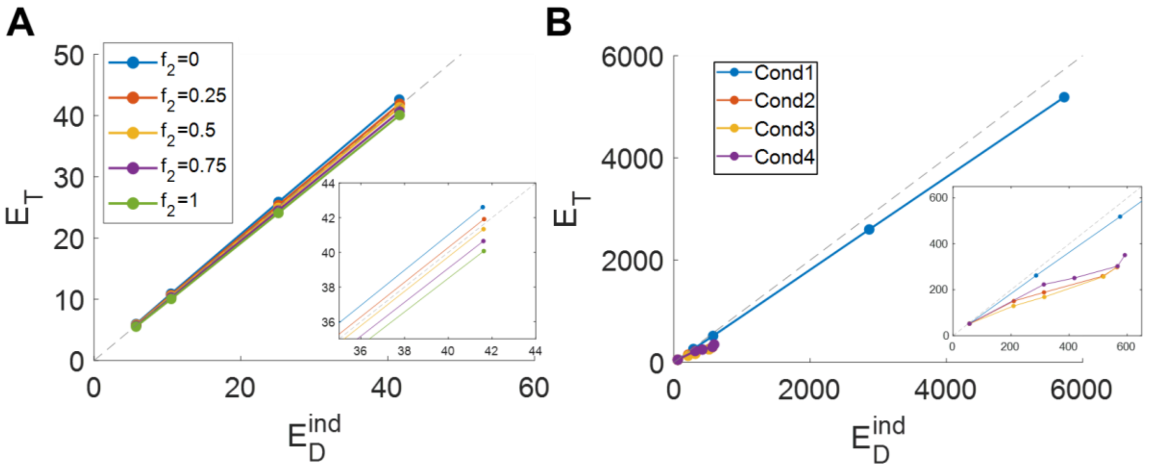

Figure S4: *In silico* expression levels of individual downstream promoters (E_D^{ind}) and non-overlapping tandem promoters (E_T). **(A)** Effects of changing the RNAP escape rate from the downstream promoter (k_{esc}^D). Each colored line represents a different relative frequency (f_2) of both RNAPs falling-off upon colliding (as opposed to only one RNAP falling-off). **(B)** Effects of changing both the rates of RNAP binding and of RNAP escape from the upstream and from downstream promoter (k_t^U, k_{esc}^U, k_t^D, and k_{esc}^D). Each colored line represents a different set of parameter values modeling different conditions. Each condition (“Cond”) is described in Table S3. The dash

line is the line of equality ($E_D^{ind} = E_T$). Finally, the insets show zoomed areas of the plots, to better distinguish which data points are above and below the dashed line.

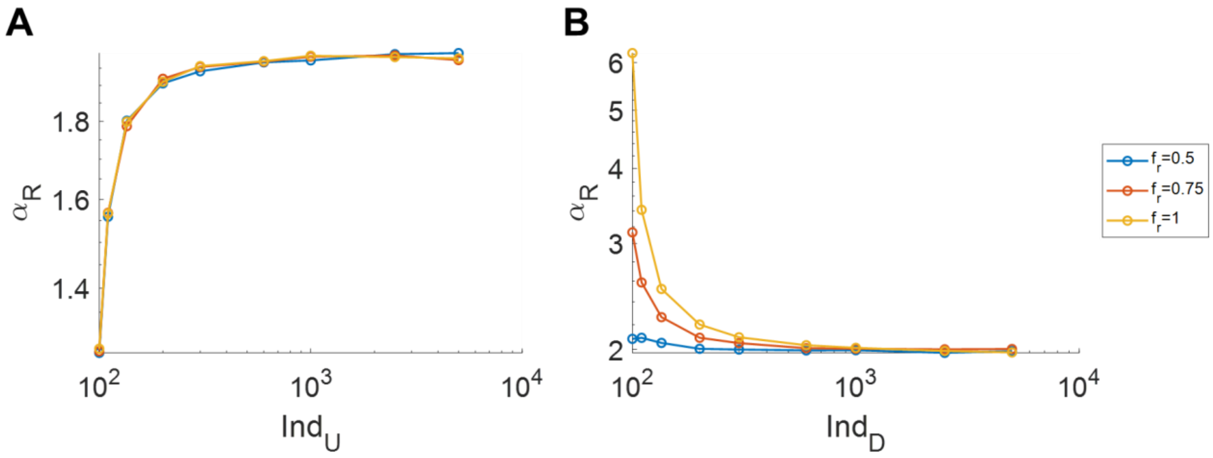

Figure S5: *In silico* estimation of α_R for non-overlapping tandem promoters when **(A)** the upstream promoter is gradually induced, and, when **(B)** the downstream promoter is gradually induced. The plots are in logarithmic scale. In (A) the downstream promoter is fully induced while in (B) the upstream promoter is fully induced. f_r stands for the relative frequency with which an RNAP from the upstream promoter falls-off upon colliding with a repressor bound to the downstream promoter (as opposed to the repressor falling-off). For f_r = 1, it is always the RNAP that falls-off with the collision.

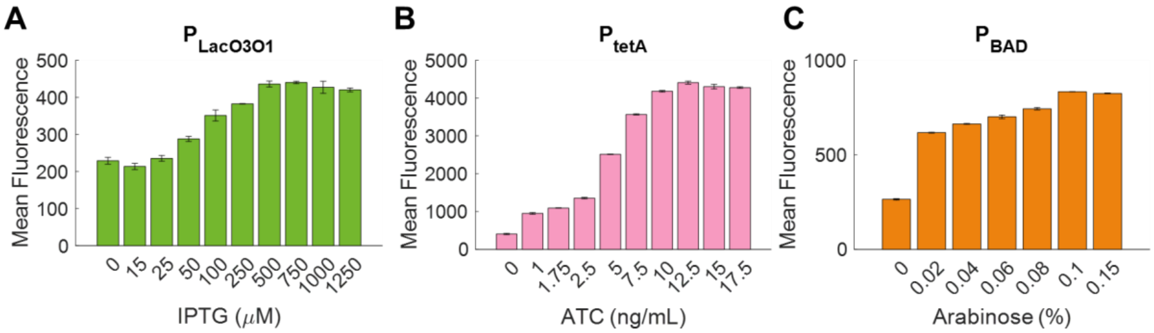

Figure S6: Induction curves of $P_{LacO3O1}$ (inducible by IPTG), P_{tetA} (inducible by aTc), and P_{BAD} (inducible by arabinose) in individual formation, as measured by single-cell fluorescence. The mean and standard error of the single-cell fluorescence were extracted from 3 biological replicates in each condition. In all cases, the strongest inducer concentration resulted in a minor decrease in fluorescence. For this reason, the previous condition was used as being "maximum induction".

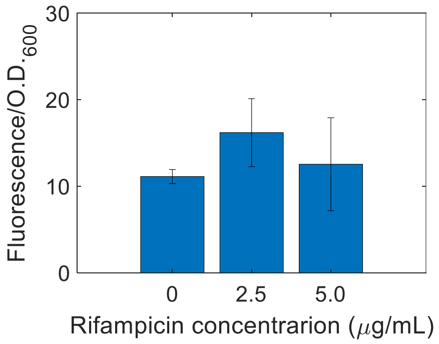

Figure S7: Fluorescence levels of DH5α-Pro cells (WT) absent of our plasmids when subject to rifampicin. Population fluorescence levels (spectrophotometry) are normalized by the corresponding O.D.$_{600}$, to be used as a proxy for average single-cell fluorescence (Methods section "Spectrophotometry"). We measured 180 minutes after adding rifampicin, when reaching O.D.$_{600}$ of 0.3. The error bars represent the SEM from 3 biological replicates.

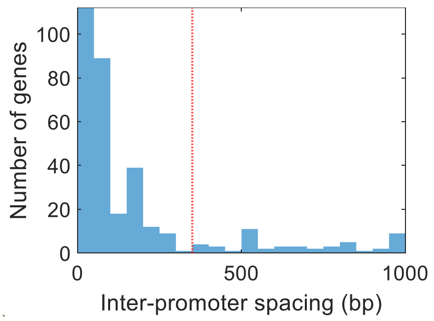

Figure S8: Distribution of inter-promoter spacing between TSSs of non-overlapping tandem promoters (in bp). The red dotted line marks the 350 bp spacing. Approximately 85% of promoters in tandem formation are spaced by less than 350 bp.

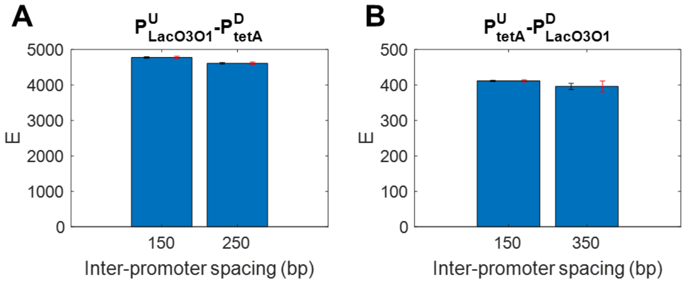

Figure S9: Mean single-cell expression levels (E) of synthetic tandem constructs differing only in inter-promoter spacing between non-overlapping tandem TSSs. The (very small) black error bars represent the standard error of the mean while the red error bars represent the standard deviation of the 3 technical replicates.

Supplementary Tables

Table S1: Parameter values of the model described in Section S1.

**Parameter**	**Value**	**Reference**
k_t^D and k_t^U	0.0013 s^{-1}	Extracted from [1]
k_{esc}^D	0.0052; 0.0104; 0.0347; 0.104 s^{-1}	Scaled to cover most of the realistic state space. The values were used to test corresponding occupancy times of 87%, 75%, 50%, and 30% of the simulation time, approximately.
k_{esc}^U	0.0052 s^{-1}	Extracted from [1]
k_{elong}	42 nucleotides/s	Extracted from [8]
No. nucleotides from the TSSs to the transcription termination site.	Pro^D: 890 nucleotides Pro^U:1030 nucleotides	Average values estimated from [4,9], using the classification of non-overlapping tandem promoters from [9].
k_c	1/3 s^{-1}	Base value of the expected time that it will take an RNAP elongating from the upstream promoter to find (if present) an RNAP bound to the downstream promoter. Estimated using k_{elong} and the average distance of 140 nucleotides between natural TSSs [9]. In our model constructs, this distance is 150 nucleotides.
f_2	0; 0.25; 0.5; 0.75; 1	
k_r^D and k_r^U	1 s^{-1}	Extracted from [10]
k_a^D and k_a^U	0.1 s^{-1}	Extracted from [10]
k_{ind}^D and k_{ind}^U	1 s^{-1}	For simplicity, Rep molecules are equally likely to bind to an inducer as to the promoter.
k_{unind}^D and k_{unind}^U	0.1 s^{-1}	
f_r	0.5; 0.75; 1	
k_d^U and k_d^D	0.00082 s^{-1}	Extracted from [11]

Table S2: Initial amounts of reactants in simulations. The initial values of all reactants not shown are set to 0.

Reactant	Initial value	Reference
RNAP	40	Extracted from [1]
Rep^U and Rep^D	100	Extracted from [10]
Ind^U and Ind^D	0; 100; 110; 135; 200; 600; 1000; 2500; 5000	Set to cover most of the realistic state space of repression strength.

Table S3: Parameter values set to obtain the plots in Figure S4B.

Condition	Description	**(k_t^U; k_t^D) (s^{-1})**	**(k_{esc}^U; k_{esc}^D) (s^{-1})**	f_2
1	Increase all rates by the same amount	(0.013; 0.013)<br>(0.065; 0.065)<br>(0.13; 0.13)<br>(0.65; 0.65)<br>(1.3;1.3)	(0.052; 0.052)<br>(0.26; 0.26)<br>(0.52; 0.52)<br>(2.6; 2.6)<br>(5.2; 5.2)	1
2	Increase k_{esc}^U and k_{esc}^D by the same amount	(0.013; 0.013)	(0.052; 0.052)<br>(0.26; 0.26)<br>(0.52; 0.52)<br>(2.6; 2.6)<br>(5.2; 5.2)	1
3	Increasing both k_{esc}^U (strong increase) and k_{esc}^D (weak increase)	(0.013; 0.013)	(0.052; 0.052)<br>(0.52; 0.26)<br>(1.04; 0.52)<br>(5.2; 2.6)<br>(10.4; 5.2)	1
4	Increasing both k_{esc}^U (weak increase) and k_{esc}^D (strong increase)	(0.013; 0.013)	(0.052; 0.052)<br>(0.26; 0.52)<br>(0.52; 1.04)<br>(2.6; 5.2)<br>(5.2; 10.4)	1

Table S4: Values of the coefficients of each of the best fittings in the main manuscript. We only present linear fits for correlations found to be statistically significant (Methods section "Fittings and statistical analysis").

Figure	Fitting formula	Coefficient values
Figure 2D	$y = \frac{C}{x} + n$	*C* = 231<br>*n* = 1.02
Figure 5A$_1$ (P_{tetA})	$y = \frac{a}{1 + e^{-b\cdot(x-c)}}$	*a* = 4386<br>*b* = 0.49<br>*c* = 4.23
Figure 5A$_1$ (P^U_{tetA}- $P^D_{LacO3O1}$)	$y = \frac{a}{1 + e^{-b\cdot(x-c)}}$	*a* = 405.5<br>*b* = 0.21<br>*c* = 0.97
Figure 5A$_2$	$y = \frac{a \cdot x + b}{x + c}$	*a* = 15.18<br>*b* = 14.04

		c = 4.34
Figure 5B$_1$ ($P_{LacO3O1}$)	$y = \frac{a}{1 + e^{-b\cdot(x-c)}}$	a = 426<br>b = 0.01<br>c = 0.08
Figure 5B$_1$ (P^{U}_{tetA}- $P^{D}_{LacO3O1}$)	$y = \frac{a}{1 + e^{-b\cdot(x-c)}}$	a = 387<br>b = 0.01<br>c = 61.14
Figure 5B$_2$	$y = \frac{x + a}{b \cdot x + c}$	a = 380.8<br>b = 0.11<br>c = 11.42
Figure 7C$_1$	$y = a \cdot x + b$	a = 0.04<br>b = 1.05
Figure 7C$_2$	$y = a \cdot x + b$	a = 4.68<br>b = 10.72
Figure 8A (f_2 = 0)	$y = \frac{a \cdot x + b}{x + c}$	a = 1.00<br>b = -0.005<br>c = 20.16
Figure 8A (f_2 = 0.25)	$y = \frac{a \cdot x + b}{x + c}$	a = 0.98<br>b = -0.02<br>c = 19.81
Figure 8A (f_2 = 0.5)	$y = \frac{a \cdot x + b}{x + c}$	a = 0.95<br>b = 0.11<br>c = 20
Figure 8A (f_2 = 0.75)	$y = \frac{a \cdot x + b}{x + c}$	a = 0.93<br>b = 0.01<br>c = 19.51
Figure 8A (f_2 = 1)	$y = \frac{a \cdot x + b}{x + c}$	a = 0.91<br>b = 0.05<br>c = 19.26
Figure 8B (f_2 = 0)	$y = a \cdot x + b$	a = 0.004<br>b = 1.06
Figure 8B (f_2 = 0.25)	$y = a \cdot x + b$	a = 0.005<br>b = 1.09
Figure 8B (f_2 = 0.5)	$y = a \cdot x + b$	a = 0.005<br>b = 1.10
Figure 8B (f_2 = 0.75)	$y = a \cdot x + b$	a = 0.005<br>b = 1.12
Figure 8B (f_2 = 1)	$y = a \cdot x + b$	a = 0.005<br>b = 1.14

Supplementary References

(1) Lloyd-Price, J., Startceva, S., Kandavalli, V., Chandraseelan, J. G., Goncalves, N., Oliveira, S. M. D., Häkkinen, A., and Ribeiro, A. S. (2016) Dissecting the stochastic transcription initiation process in live Escherichia coli. *DNA Res. 23*, 203–214.

(2) deHaseth, P. L., Zupancic, M. L., and Record, M. T., Jr. (1998) RNA polymerase-promoter interactions: the comings and goings of RNA polymerase. *J. Bacteriol. 180*, 3019–3025.

(3) Hsu, L. M. (2002) Promoter clearance and escape in prokaryotes. *Biochimica et Biophysica Acta (BBA) - Gene Structure and Expression 1577*, 191–207.

(4) Tierrafría, V. H., Rioualen, C., Salgado, H., Lara, P., Gama-Castro, S., Lally, P., Gómez-Romero, L., Peña-Loredo, P., López-Almazo, A. G., Alarcón-Carranza, G., Betancourt-Figueroa, F., Alquicira-Hernández, S., Polanco-Morelos, J. E., García-Sotelo, J., Gaytan-Nuñez, E., Méndez-Cruz, C.-F., Muñiz, L. J., Bonavides-Martínez, C., Moreno-Hagelsieb, G., Galagan, J. E., Wade, J. T., and Collado-Vides, J. (2022) RegulonDB 11.0: Comprehensive high-throughput datasets on transcriptional regulation in Escherichia coli K-12. *Microb Genom 8*.

(5) Mäkelä, J., Lloyd-Price, J., Yli-Harja, O., and Ribeiro, A. S. (2011) Stochastic sequence-level model of coupled transcription and translation in prokaryotes. *BMC Bioinformatics 12*, 121.

(6) Jagadeesan, R., Dash, S., Palma, C. S. D., Baptista, I. S. C., Chauhan, V., Mäkelä, J., and Ribeiro, A. S. (2023) Dynamics of bacterial operons during genome-wide stresses. *bioRxiv* 2023.08.24.554578.

(7) Ma, J., Bai, L., and Wang, M. D. (2013) Transcription under torsion. *Science 340*, 1580–1583.

(8) Proshkin, S., Rahmouni, A. R., Mironov, A., and Nudler, E. (2010) Cooperation between translating ribosomes and RNA polymerase in transcription elongation. *Science 328*, 504–508.

(9) Chauhan, V., Bahrudeen, M. N. M., Palma, C. S. D., Baptista, I. S. C., Almeida, B. L. B., Dash, S., Kandavalli, V., and Ribeiro, A. S. (2022) Analytical kinetic model of native tandem promoters in E. coli. *PLoS Comput. Biol. 18*, e1009824.

(10) Yu, J., Xiao, J., Ren, X., Lao, K., and Xie, X. S. (2006) Probing gene expression in live cells, one protein molecule at a time. *Science 311*, 1600–1603.

(11) Chen, Shiroguchi, K., Ge, H., and Xie, X. S. (2015) Genome-wide study of mRNA degradation and transcript elongation in Escherichia coli. *Mol. Syst. Biol. 11*, 781.
